# Synthetically recoded virus sCPD9 – A tool to accelerate SARS-CoV-2 research under biosafety level 2 conditions

**DOI:** 10.1016/j.csbj.2022.08.027

**Published:** 2022-08-13

**Authors:** Dusan Kunec, Nikolaus Osterrieder, Jakob Trimpert

**Affiliations:** a Institut für Virologie, Freie Universität Berlin, Berlin, Germany; b Department of Infectious Diseases and Public Health, Jockey Club College of Veterinary Medicine and Life Sciences, City University of Hong Kong, Kowloon, Hong Kong

**Keywords:** Modified live virus vaccine, SARS-CoV-2, Large-scale genome recoding, Codon pair deoptimization, Biosafety level 2, Viral diagnostics, Antiviral development

## Abstract

Research with infectious SARS-CoV-2 is complicated because it must be conducted under biosafety level 3 (BSL-3) conditions. Recently, we constructed a live attenuated SARS-CoV-2 virus by rational design through partial recoding of the SARS-CoV-2 genome and showed that the attenuated virus, designated sCPD9, was highly attenuated in preclinical animal models. The recoded sequence was designed by codon pair deoptimization and is located at the distal end of gene ORF1ab. Codon pair deoptimization involves recoding of the viral sequence with underrepresented codon pairs but without altering the amino acid sequence of the encoded proteins. Thus, parental and attenuated viruses produce exactly the same proteins. In Germany, the live attenuated SARS-CoV-2 mutant sCPD9 was recently classified as a BSL-2 pathogen based on its genetic stability and strong attenuation in preclinical animal models. Despite its high attenuation *in vivo*, sCPD9 grows to high titers in common cell lines, making it suitable as substitute for virulent SARS-CoV-2 in many experimental setups. Consequently, sCPD9 can ease and accelerate SARS-CoV-2 research under BSL-2 conditions, particularly in experiments requiring replicating virus, such as diagnostics and development of antiviral drugs.

## 1 Introduction

Severe Respiratory Syndrome Coronavirus 2 (SARS-CoV-2) can only be studied under BSL-3 conditions [1]. This complicates and restricts research with this pathogen to institutions that operate high containment biological laboratories. The limited capacity, restricted access, and high costs of these laboratories preclude many researchers from working with SARS-CoV-2, which limits scientific exploration and delays better understanding of the virus and the many disease manifestations it causes.

Here, we present sCPD9, a replication-competent and attenuated SARS-CoV-2 vaccine candidate [2], [3], which has recently been approved for use under BSL-2 conditions in Germany. The sCPD9 virus was generated through codon pair deoptimization (CPD), also known as synthetic attenuated virus engineering (SAVE), a strategy that has enabled rapid and highly efficient attenuation of a wide variety of RNA viruses, including Enterovirus C (poliovirus) [4], Influenza A virus (IAV) [5], Human immunodeficiency virus type 1 [6], Human respiratory syncytial virus [7], Indiana vesiculovirus [8], and Dengue virus [9]. Although CPD has been used primarily to attenuate RNA viruses, it is also applicable for DNA viruses [10], [11] and bacteria [12]. In CPD, viral genes are extensively recoded/rewritten, but the amino acid sequence of the encoded proteins is not changed [4]. Attenuation by CPD is based on the observation that some codon pairs in protein-coding sequences are significantly less or more abundant than statistically expected [4]. CPD introduces hundreds of point mutations into viral sequences by rearranging the positions of synonymous codons, while preserving the amino acid composition of the encoded protein(s) [4], [13]. The goal of CPD is to design viral genes with an increased number of codon pairs that are statistically underrepresented (suboptimal) in the host genome, because underrepresented codon pairs reduce protein production of the recoded genes and may be more potent stimulators of innate immune responses by altered nucleotide composition of encoded RNA [4], [14]. Consequently, codon pair-deoptimized viruses are attenuated but the preserved antigenic identity and replication capacity allow attenuated viruses to elicit strong immune responses that are virtually identical to those of wild-type viruses. These properties make codon pair deoptimized viruses not only attractive vaccine candidates, but also useful tools to study pathogens in an attenuated setting and, thus, under less restrictive biosafety conditions.

A prerequisite for recoding by CPD is knowledge regarding the codon pair biases observed in naturally occurring codon pair combinations. The goal of recoding by CPD is to increase the occurrence of codon pairs that are naturally underrepresented in host protein coding sequences. The recoding algorithm must therefore be able to assess which codon swaps reduce the overall codon pair bias of the recoded sequence and which do not. Codon pair bias of each codon pair can be calculated as codon pair score (CPS) [4]. The CPS of a codon pair is defined as the natural logarithm of the ratio between the observed (actual) number of codon pair occurrences and the expected number of codon pair occurrences in the analyzed protein-coding sequences. The exact codon pair bias of a species is calculated based on all protein-coding sequences, i.e., the complete set of ORFs in the genome of a species (species’ ORFeome). A positive CPS value indicates that the particular codon pair is statistically overrepresented (more frequent) in the analyzed ORFeome compared with what would be expected based on the individual frequencies of two codons in the ORFeome that form the given codon pair [4]. Similarly, a negative CPS value indicates that the codon pair is statistically underrepresented in the analyzed ORFeome [4].

To generate SARS-CoV-2 mutants that would be attenuated in humans, we recoded the SARS-CoV-2 genome using the CPS calculated for each of the 3,721 codon-pair combinations (61 × 61 codons, excluding stop codons) based on all representative human protein-coding genes [15]. Since there is a large number of ways how a given protein can be encoded, recoding a gene to the maximum level of codon pair deoptimization is computationally difficult and time consuming. An approximate, near-optimal solution to this problem, which is more than sufficient for attenuation purposes, can be quickly found using heuristic or metaheuristic approaches. Our recoding program [10], similar to the algorithm of Coleman et al. [4], uses simulated annealing, a fast metaheuristic algorithm, to find a good approximation to the absolute codon pair bias minimum [16].

**Fig. 1:**
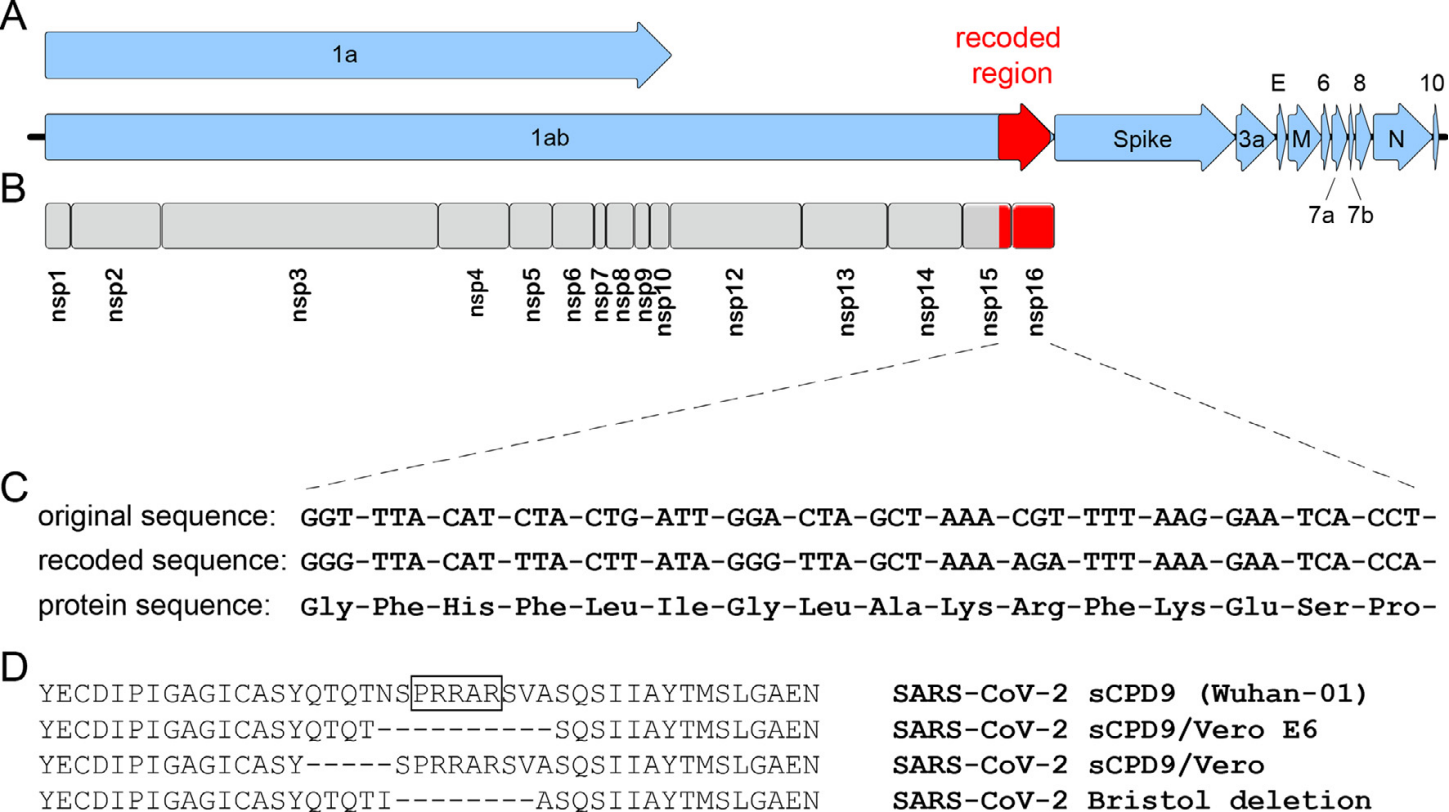
The genetic structure of the live attenuated SARS-CoV-2 mutant sCPD9. (A) The SARS-CoV-2 genome is a single-stranded, positive-sense mRNA molecule of approximately 30,000 nt, which encodes 11 canonical ORFs (blue arrows). (B) After infection, ORF 1a/1ab is directly translated and cleaved into 15 non-structural proteins (nsp) that form the replication-transcription complex. The genome of sCPD9 virus contains a recoded, codon pair deoptimized sequence of 1,146 nt in length (red segment of the ORF 1ab). The recoded sequence encodes parts of nsp15 – an endoribonuclease and nsp16 – a 2′O-ribose methyltransferase. (C) Exemplary depiction of the first 16 codons of the original sequence of the SARS-CoV-2 genome that was replaced with the corresponding recoded sequence. Note that the original and recoded sequences encode identical amino acids. D) Deletions identified in spike glycoprotein after passage in cultured cells. An alignment of spike protein sequences from the region that contains the furin cleavage site. The amino acid residues constituting the furin cleavage site are marked by a box in the first lane. The “Bristol deletion” is a deletion that occurred naturally during serial passage of SARS-COV-2 in cell culture [19] it is shown here for comparison. (For interpretation of the references to colour in this figure legend, the reader is referred to the web version of this article.)

The sCPD9 virus was constructed using a reverse genetics system of SARS-CoV-2 [17]. The system is based on 12 subgenomic fragments of the SARS-CoV-2 genome, which are assembled into a single yeast/bacterial artificial chromosome (YAC/BAC) by transformation-associated recombination (TAR) cloning in *Saccharomyces cerevisiae* [18]. The YAC/BAC clone containing a copy of the complete SARS-CoV-2 genome was then transferred into E. coli, where the SARS-CoV-2 genome was modified by tools of bacterial genetics [3]. To generate sCPD9, a short segment of the parental SARS-CoV-2 genome was as removed and replaced with a recoded, synthetically produced DNA sequence. Specifically, a 1146 nt sequence in the SARS-CoV-2 gene ORF1ab (nucleotide positions 20,359–21,504, GenBank: MT108784) was replaced with a codon pair-deoptimized sequence (Fig. 1A–C). The recoded sequence encodes the SARS-CoV-2 proteins nsp15 (endoribonuclease) and nsp16 (2′O-methyltransferase). Thus, except for the recoded region, the parental and sCPD9 viruses contain the same genomic sequence and encode the exact same proteins. *In vitro* growth kinetics demonstrated stable replication of sCPD9 to titers about 10-fold lower compared to parental wild type [3]. sCPD9 was further characterized in animal experiments involving Syrian and Roborovski dwarf hamsters, where it showed a high degree of attenuation while in the same time maintaining the ability to induce protective immunity [2], [3].

## 2 Results

One of the most important criteria for live attenuated virus vaccines is that they are genetically stable and thus unable to revert to their pathogenic phenotype and cause disease. This risk particularly exists when only a few nucleotide changes are responsible for the attenuation, as was the case with the oral poliovirus vaccine [20]. Viruses attenuated by CPD are considered genetically stable because their attenuation is due to additive defects caused by hundreds of introduced nucleotide mutations [4]. Genetic stability of an attenuated virus is a prerequisite for approval of a replicating SARS-CoV-2 for use under BSL-2 conditions, considering that the attenuation was attained through genetic recoding.

**Fig. 2:**
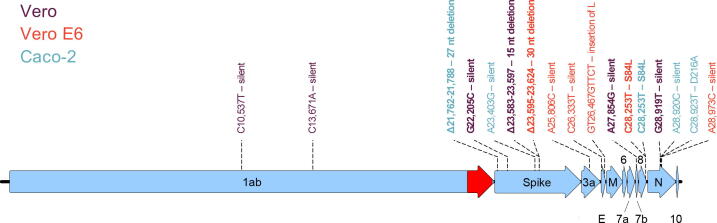
Mutations detected in sCPD9 viral populations after 10 serial passages in Vero, Vero E6, and Caco-2 cells. Mutations that were detected in virus populations with a frequency higher than 50% are shown in bold. The colors indicate the cells in which virus populations harboring specified mutations were serially propagated.

To determine the genetic stability of sCPD9 virus in cell culture, we passaged sCPD9 ten times in African green monkey Vero, Vero E6 or human intestinal epithelial Caco-2 cells. Sequencing showed that limited serial passage did not significantly alter the structure of the sCPD9 virus genome, as no single nucleotide polymorphisms (SNP), insertions or deletions were detected within the recoded sequence after 10 passages on Vero, Vero E6 or Caco-2 cells (Fig. 2, Table 1). As expected, and also observed for wild type viruses [19], [21], passaging resulted in the accumulation of a small number of SNPs distributed throughout the viral genome and the selection of viral variants containing small, 15–30 nt in-frame deletions in the spike gene (Fig. 2, Table 1).

**Table 1:** Mutations detected in viral populations after 10 serial passages in Vero, Vero E6, and Caco-2 cells. The upper part of the table shows mutations detected in virus populations with a frequency higher than 50%, the lower part those with a frequency higher than 10%. Numbers indicate genomic position of detected mutations. Based on computed quality scores, numbers in bold indicate a high probability of detected mutations being real, while numbers in normal font indicate a lower probability that the mutations were correctly identified.

Locus	Nucleotide position			Mutation type	Mutation (%)
	Vero	Vero E6	Caco-2		
*Mutations with a penetrance of more than 50%*					
S			**21,762**–**21,788**	27 nt deletion	100.0
S	**22,205**			G → C; silent	91.8
S	**23,583**–**23,597**			15 nt deletion	93.0
S		**23,595**–**23,624**		30 nt deletion	100.0
ORF7b	**27,854**			A → G; silent	80.7
ORF8		**28,253**		C → T; S84L	100.0
ORF8			**28,253**	C → T; S84L	85.4
N	**28,919**			G → T; silent	95.5

*Mutations with a penetrance of more than 10%*					
nsp5	10,537			C → T; silent	11.1
RdRp	13,671			C → A; silent	15.4
S			**23,403**	A → G; silent	35.5
ORF3a		25,806		A → C; silent	38.0
E		26,333		C → T; silent	31.7
E		26,467–26,468		GT → GTTCT; ins. L	12.9
N			**28,920**	A → C; silent	40.6
N			**28,923**	C → T; D216A	33.1
N		28,973		A → C; silent	16.3

Most sCPD9 virus variants that emerged from the propagation on Vero E6 cells contained a 10 amino acids deletion (NSPRRARSVA) in the spike protein that encompasses the entire furin cleavage site. Similarly, sCPD9 virus populations that originated from the passage on Vero cells contained a 15 nt deletion encoding 5 amino acids ‘QTQTN’ located directly upstream of the furin cleavage site (Table 1). These results are consistent with previous reports showing that SARS-CoV-2 variants lacking a functional furin cleavage site become rapidly dominant when SARS-CoV-2 is propagated in Vero or Vero E6 cells which do not constitutively express TMPRSS2 [22]. In such TMPRSS2-deficient cells, variants lacking a functional furin cleavage site appear to have a strong selective advantage [21]. However, loss of the furin cleavage site is expected to further attenuate SARS-CoV-2 [23], so the use of mutants lacking the furin cleavage site is not considered problematic under BSL-2 conditions. When sCPD9 was propagated on Caco-2 cells, which express moderate levels of TMPRSS [22], the virus did not lose the furin cleavage site but acquired a 27 nt deletion within the *N*-terminal domain of the spike protein (Table 1). Because sCPD9 grows only to relatively low titers on Caco-2 cells, and sCPD9 was genetically unstable during its propagation on these cells, we do not consider Caco-2 cells to be an optimal choice for propagation of sCPD9.

We applied to the German Central Committee on Biological Safety (Zentrale Kommission für Biologische Sicherheit, ZKBS) for reclassification of the attenuated SARS-CoV-2 mutant sCPD9 from BSL-3 to BSL-2 based on its biological properties, in particular its near-complete attenuation in preclinical animal models and its genetic stability under limited passage in cultured cells. Our application was positively evaluated, and the SARS-CoV-2 sCPD9 with and without the furin cleavage site is now classified as a BSL-2 organism in Germany.

## 3 Conclusions

We contend that downgrading of the attenuated virus sCPD9 sets an important precedent. We anticipate that reclassification of attenuated SARS-CoV-2 viruses to BSL-2, as in the case of sCPD9, will democratize, simplify, and expedite SARS-CoV-2 research that requires the use of replicating virus, especially but not only at institutions that only operate BSL-2 laboratories. In addition to the recent ZKBS recommendation for BSL-2 classification of sCPD9 in Germany, sCPD9 was recently downgraded to BSL-2 level in the Netherlands and a similar application is currently under evaluation by the Swiss Expert Committee for Biosafety (SECB).

The sCPD9 virus, and its future derivatives, could prove to be particularly valuable tools in virus diagnostics, for example for determining virus neutralization titers. The sCPD9 virus could also be used for (high-throughput) screening of neutralizing antibodies or antiviral compounds. In addition, we envision that the sCPD9 virus could be also very useful in basic research to study biological questions that do not need to be addressed with a pathogenic virus. Moreover, the ability to produce virus under BSL-2 conditions greatly facilitates vaccine production, both in the case of a live attenuated vaccine and vaccines based on inactivated virus particles.

The major limitation of sCPD9 virus for many potential applications, such as viral diagnostics or screening of neutralizing antibodies, is that its genome is that of the ancestral viral variant Wuhan-01 [3]. However, because sCPD9 was generated using a facile reverse genetics system of SARS-CoV-2 [17], sCPD9 can be rapidly modified to contain genetic sequences of different SARS-CoV-2 variants. Here, the spike protein as major surface antigen of SARS-CoV-2 and rapidly evolving target of immunity and therapeutic interventions, is in the focus of attention. Because the attenuating mutation in sCPD9 is located at the end of ORF1ab, the defining antigenic properties of sCPD9 could be adapted to those of other strains by exchanging the spike gene. Thus, the attenuated virus could be adapted to contain a spike protein to meet the need of different diagnostic or therapeutic strategies.

Alternatively, for applications that require an intact antigenic repertoire, SARS-CoV-2 variants can become attenuated through insertion of the sCPD9 mutations into their genome. We are currently generating SARS-CoV-2 variants Delta and Omicron BA.2 that would carry the same attenuating mutation, and we expect to expand the number of SARS-CoV-2 mutants that can be studied under BSL-2 conditions soon.

## 4 Material and methods

### 4.1 Cell culture of Vero, Vero E6 and Caco-2 cells

African green monkey Vero (WHO RCB 10–87), Vero E6 cells (ATCC CRL-1586) or human intestinal epithelial Caco-2 (ATCC HTB-37) cells were grown in minimal essential medium (MEM) containing 10% fetal bovine serum (PAN Biotech), 100 IU/ml penicillin G and 100 µg/ml streptomycin at 37 °C and 5% CO_2_.

### 4.2 Transfection of Vero E6 cells with SARS-CoV-2 BAC DNA

To recover infectious sCPD9 virus, Vero E6 cells were grown to 70% confluence in a T25 cell culture flask and transfected with 4 µg of BAC DNA and 1 µg of helper plasmid pVITRO2-EGFP-N using the Xfect single shots transfection reagent (Takara Bio). The transfected cells were incubated for 72 h, then the cell culture medium containing the recovered virus was transferred to fresh Vero E6 cells to create passage 1 stocks of recovered virus.

### 4.3 Serial passage of sCPD9 in cultured cells

Upon development of visible cytopathic effects, virus stocks were frozen and titrated by plaque assay. To initiate passaging, Vero, Vero E6, or Caco-2 cells were infected with an MOI of 0.05. After 48 h, infected cells were lysed by a freeze–thaw cycle and 1% of the supernatant and cell lysate was used as inoculum for the next passage, this procedure was repeated 10 times in total.

### 4.4 High-throughput sequencing and data analysis

The genomic sequence of sCPD9 virus populations after serial passage was determined by next-generation sequencing on the Illumina MiSeq platform.

To sequence viral genomes, total RNA was isolated from infected cells using Trizol reagent (Thermo Fisher Scientific) according to the manufacturer’s instructions. Sequencing libraries were constructed using the NEBNext Ultra II RNA kit (New England Biolabs) with NEBNext Multiplex Oligos for Illumina (New England Biolabs).

The obtained sequencing reads were processed with Trimmonatic v.0.36 and mapped against the genomic sequence of sCPD9 virus using the Burrows-Wheeler aligner, version 0.7.15. Single nucleotide polymorphisms (SNP) as well as insertions or deletions (indel) were evaluated using FreeBayes version 1.1.0–333. Data were merged by position and mutation using R v.3.2.3. The SNPs were additionally assessed using Geneious R11 software (Biomatters).

## CRediT authorship contribution statement

**Dusan Kunec:** Conceptualization, Methodology, Software, Investigation, Writing – review & editing, Visualization. **Nikolaus Osterrieder:** Conceptualization, Writing – original draft, Writing – review & editing, Funding acquisition. **Jakob Trimpert:** Conceptualization, Methodology, Investigation, Writing – original draft, Writing – review & editing, Supervision, Project administration, Funding acquisition.

## Declaration of Competing Interest

Related to this work, Freie Universität Berlin has filed a patent application for the use of sCPD9 as vaccine. In this application, JT, NO and DK are named as inventors of sCPD9. Freie Universität Berlin is collaborating with RocketVax Inc. for further development of sCPD9 as vaccine and receives funding for research.
